# Alisol A 23,24-diacetate improves LPS-induced acute lung injury through the IL-17A/NF-κB signaling pathway

**DOI:** 10.1186/s13020-026-01487-w

**Published:** 2026-08-24

**Authors:** Yuhan Zhang, Pengli Guo, Mengnan Zeng, Denghui Zhu, Ziyu Zhang, Mengdie Luo, Yi Chao, Fangzhuo Chang, Weisheng Feng, Xiaoke Zheng

**Affiliations:** 1 College of Pharmacy, Henan University of Chinese Medicine, Zhengzhou, China; 2 The Engineering and Technology Center for Chinese Medicine Development of Henan Province, Zhengzhou, China

**Keywords:** Alisma orientale, Alisol A 23,24-diacetate, Lung organoids, Zebrafish, IL-17A/NF-κB

## Abstract

**Objective:**

This study utilized the multi-model validation method to confirm that Alisol A 23,24-diacetate (AAD) can improve LPS-induced acute lung injury (ALI) through the IL-17A/NF-κB signaling pathway, providing experimental data support for preclinical research.

**Methods:**

Mice were given drugs by gavage continuously for 3 days, and then LPS was instilled into trachea to establish the model of acute lung injury. Subsequently, the lung pathological sections, lung function, the levels of oxidative stress and apoptosis, the metabolic levels in lung tissue and serum, and the levels of immune cells and inflammatory factors in each group of mice were detected. Then, the differentially expressed genes were screened based on the GEO database, and molecular docking combined with PCR technology was employed to investigate the potential pathogenesis of acute lung injury. Next, in vivo mouse models and in vitro lung organoid models were utilized, combined with Western blot and immunofluorescence techniques, to validate the proteins associated with the IL-17A/NF-κB signaling pathway. The binding of AAD to IL-17A protein was detected by cellular thermal shift assay (CETSA). Finally, the Beas-2B cell model combined with IL-17 agonist (SR0987) and IL-17 antagonist (Brodalumab), as well as the zebrafish model coupled with the methods of IL-17A gene silencing and overexpression, were employed to validate the mechanism of action underlying the intervention of AAD in LPS-induced ALI.

**Results:**

AAD significantly ameliorated lung injury and lung function in a mouse model of LPS-induced ALI, modulated metabolic and immune disorders in ALI mice, and suppressed the release of inflammatory factors. The results from GEO database analysis, molecular docking, PCR, WB, immunofluorescence and CETSA, demonstrated that AAD ameliorates LPS-induced ALI via the IL-17A/NF-κB signaling pathway. In Beas-2B cells, AAD significantly suppressed the LPS-induced elevation of ROS and apoptosis levels, reduced the protein expression of IL-17A and p-NF-κB p65, and decreased the levels of inflammatory factors, exerting a comparable therapeutic effect to that of the IL-17 antagonist. However, the therapeutic efficacy of AAD was abrogated following the addition of an IL-17 agonist. In the experiment of zebrafish, AAD significantly enhanced the sports activity of zebrafish and inhibited the levels of IL-17A and inflammatory factor RNA. However, the therapeutic efficacy of AAD was abrogated following IL-17A gene overexpression.

**Conclusion:**

AAD improves LPS-induced ALI through the IL-17A/NF-κB signaling pathway.

**Supplementary Information:**

The online version contains supplementary material available at 10.1186/s13020-026-01487-w.

## Introduction

The lungs are a crucial respiratory organ in the human body, responsible for gas exchange, maintaining the body's oxygen balance, and defending against pathogen invasion, among other core functions [1]. Lung injury can be triggered by various factors, among which acute lung injury (ALI) induced by lipopolysaccharide (LPS) is a common critical and urgent condition in clinical practice [2, 3], often following severe infections, trauma, and sepsis, and has become a global health threat [4]. In its advanced stage, it can rapidly progress to acute respiratory distress syndrome (ARDS), with a mortality rate as high as 30–50% [5, 6]. Although the pathological mechanism of ALI has been studied in depth, there are still no targeted effective therapeutic drugs in clinical practice. Mainly relying on supportive treatments such as mechanical ventilation and fluid management, the treatment effect is limited [7]. To date, various natural Chinese herbal medicines have shown significant anti-inflammatory, antioxidant, and organ-protective effects in clinical practice [8, 9], providing a new direction for the treatment of inflammatory diseases. This indicates that the active components in natural Chinese herbal medicines are an important material basis for developing ALI treatment drugs.

Alisma orientale is the dried tuber of Alisma orientale (Sam.) *Juzep.* and *Alisma planta go-aquatica Linn.*, and it has the functions of promoting diuresis, reducing dampness, clearing heat and promoting urination [10]. Modern pharmacological studies have shown that the components of Alisma orientale mainly include triterpenoids, diterpenoids, sesquiterpenoids, phenylpropanoids, flavonoids, steroids and nitrogen-containing compounds, which have significant pharmacological activities such as anti-inflammatory, antioxidant and anti-fibrosis [11, 12]. It suggests that *Alisma orientale* may have a protective effect on ALI. Previous laboratory research has isolated a large number of triterpenoid compounds from Alisma orientale, which can significantly improve Beas-2B cell damage [13–15]. Among them, alisol A 23,24-diacetate (AAD) is a triterpenoid compound isolated and identified from the dried tuber of Alisma orientale. The laboratory has previously extracted and isolated sufficient monomers for subsequent experiments. Moreover, the pharmacological experiments on this compound are currently still unexplored, so choosing AAD as the research subject is quite innovative and can further enrich the pharmacological activities of *Alisma orientale*.

Numerous studies have shown that the dysregulation of inflammatory signaling pathways is the core pathological mechanism of LPS-induced ALI [16, 17]. LPS can directly induce the production of IL-17A. When IL-17A binds to its receptor, it can further activate NF-κB, stimulating the secretion of inflammatory factors, thereby accelerating the development of ALI [18, 19]. Thus, it can be seen that IL-17A/NF-κB signaling pathway plays a crucial role in ALI. Therefore, this study constructed various models of ALI induced by LPS, and conducted in-depth research on the improvement effect of AAD on ALI and the related molecular mechanisms. The results of this study can provide a theoretical basis for the in-depth development and utilization of *Alisma orientale*, and also offer new candidate compounds and action mechanisms for the development of therapeutic drugs for ALI.

## Materials and methods

### Extraction and isolation

The tubers of *Alisma orientale* were collected from Jiyang Town, Fujian Province, China in June 2022. It was identified as the dried tubers of *Alisma orientale* by Professor Chengming Dong from the School of Pharmacy, Henan University of Chinese Medicine. A voucher specimen (DFZX20220618) is deposited at the Henan Engineering and Technology Research Center for Traditional Chinese Medicine Development. The dried tubers were extracted twice with 50% acetone by tissue homogenization. The combined extract was sequentially partitioned with petroleum ether, dichloromethane, ethyl acetate, and n-butanol, yielding five fractions: petroleum ether fraction, dichloromethane fraction, ethyl acetate fraction, n-butanol fraction, and aqueous fraction. The dichloromethane fraction was subjected to silica gel column chromatography and eluted with a gradient of dichloromethane: methanol (100:1, 80:1, 30:1, 15:1, 8:1, 5:1, 3:1, 2:1, 1:1), affording 11 subfractions (Fr. 1–11). Fr. 5 was further purified by Sephadex LH-20 CC with 70% methanol as the eluent under isocratic conditions, giving 9 subfractions (Fr. 5-1 to 5–9). Fr. 5–4 was chromatographed on Sephadex LH-20 CC using 50% methanol for isocratic elution, resulting in 4 subfractions (Fr. 5-4-1 to 5-4-4). Finally, Fr. 5-4-2 was isolated and purified by semi-preparative high-performance liquid chromatography with a mobile phase of acetonitrile–water (60:40) at a flow rate of 3 mL/min, yielding alisol A 23,24-diacetate (AAD), *t*_R_ = 33.7 min. The detailed identification process is provided in S1.

### Chemicals and reagents

Resveratrol (S30630) was purchased from Yuanye Biotechnology Co., Ltd. (Shanghai, China). Lipopolysaccharide (LPS, L2880) was purchased from Sigma‒Aldrich Trading Co., Ltd. (St. Louis, MO, USA).

### Animals

Male BALB/c mice were purchased from Beijing Viton Lihua Laboratory Animal Technology Co. Ltd (SCXK(jing)2021-0006). All animal experiments were approved by the Ethical Committee of the Henan University of Chinese Medicine, China (DWLL2018080003).In Experiment, mice were divided into five groups: Control (Con), Model (M), Resveratrol (30 mg/kg), low-dose AAD (AAD-L, 10 mg/kg) and high-dose AAD (AAD-H, 20 mg/kg), with 10 mice per group. The drug was given by gavage for 3 days. After the drug was given, LPS (5 mg/kg) was injected into the trachea for modeling in all groups except the normal group [20]. The methods for collecting serum and broncho-alveolar lavage fluid (BALF) are as described in the literature [21].

### Cell culture

Beas-2B was grown in DMEM supplemented with 10% FBS and was cultured in a 5% CO₂ incubator at 37 °C. Cells were seeded when they were in the logarithmic growth phase. The cells were divided into the normal group, model group (LPS, 10 ng/mL), AAD group (10 µM), as well as groups treated with IL-17A agonist (SR0987, 10 µM) and antagonist (Brodalumab, 10 µg/mL) respectively. Pretreatment with the agonist and inhibitor was conducted 2 h before administration. Detection of relevant indicators was performed 24 h after drug administration.

### Lung organoids culture

Primary lung organoids are extracted using a kit (Absin, abs9515). Fresh lung tissues were placed in a biosafety cabinet, rinsed 3 times with pre-chilled PBS buffer, and cut into small tissue pieces of 1–2 mm^3^. Subsequently, the tissue pieces were transferred to a centrifuge tube containing digestive solution (abs9515), and digested with shaking in a 37 ℃ constant-temperature shaker for 30 min. During digestion, the centrifuge tube was gently inverted every 10 min to ensure sufficient digestion. Following digestion, an equal volume of medium was added to terminate the digestion process. The mixture was filtered through a 100 μm cell strainer, and the filtered cell suspension was centrifuged at 1200 rpm for 5 min. The supernatant was discarded, and the cells were resuspended and washed once with pre-chilled PBS buffer. The washed cell pellet was resuspended in melted Matrigel, and 20 μL of the mixture was uniformly dropped onto the bottom of a 24-well culture plate (1 drop per well). The plate was placed in an incubator for 15 min to allow solidification, after which 500 μL of complete medium (abs9515) was added to each well. The plate was then cultured in a 37 ℃ incubator with 5% CO₂. Experiments were performed when the lung organoids grew to a diameter of 200–300 μm with intact morphology and clear internal structures. The organoids were divided into three groups: normal group, model group, and drug administration group. After 48 h of intervention, the supernatant and lung organoids were collected for subsequent experiments.

### Zebrafish

Wild-type (AB strain) zebrafish were raised in a cycle of 14 h of light and 10 h of darkness, with a water temperature of around 28 ℃. Healthy embryos were collected after the natural mating of sexually mature zebrafish and then cultivated in E3 medium. Three-day post-fertilization (dpf) zebrafish larvae were randomly divided into 9 groups: control group (Con), model group (M, LPS, 100 µg/mL), AAD group (AAD, LPS, 100 µg/mL + AAD, 10 µM), sgRNA control group (Si-Con), sgRNA silencing model group (Si-M, LPS, 100 µg/mL), sgRNA silencing administration group (sg-AAD, LPS, 100 µg/mL + AAD, 10 µM), overexpression control group (Ov-Con), overexpression model group (Ov-M, LPS, 100 µg/mL), and overexpression administration group (Ov-AAD, LPS, 100 µg/mL + AAD, 10 µM). Each group was set with 3 biological replicates, containing 50 larvae per replicate. The immersion administration method was adopted, and related indicators were detected after 24 h of intervention.

### Injection of sgRNA and overexpression plasmids

The sgRNA and overexpression plasmids were synthesized by Shanghai Feixi Biotechnology Co., Ltd. The synthesized sequences are shown in S3.

For sgRNA: 1 μL of each sgRNA, 1 μL of Cas9 protein, and 7 μL of nuclease-free water were mixed thoroughly, incubated at 37 ℃ for 15 min, and then microinjected into zebrafish embryos with an injection volume of  L per embryo.

For overexpression plasmids: The plasmids were diluted to a final concentration of 400 ng/μL prior to injection, with 2 nL injected per embryo. Twenty-four hours after injection, the efficiency of gene silencing and overexpression was verified by PCR.

### Hematoxylin and eosin (H&E) staining

Lungs: Take the pulmonary lobe and immerse it in 3.7% paraformaldehyde, and then dehydrated through a graded ethanol series. After paraffin embedding, 5 μm thick sections were prepared. Histopathological changes were assessed using HE staining.

Lung organoids: The lung organoids were isolated from the matrix gel and fixed with 3.7% polyformaldehyde, and then dehydrated through a graded ethanol series. After paraffin embedding, 3 μm thick sections were prepared. Histopathological changes were assessed using HE staining.

Zebrafish: Fix the zebrafish in 3.7% polyformaldehyde 24 h after drug intervention, and then dehydrated through a graded ethanol series. Follow the same procedure as above.

### Measurement of lung coefficient, lung water content and wet-dry ratio

The lung tissue was rinsed with PBS solution, dried on filter paper, and weighed. The lung index = (lung weight/body weight) × 100%. Five mice from each group were selected for each lung tissue sample. First, the weight was measured (lung wet weight), then the sample was dried in an oven, and the weight was measured again (lung dry weight). The lung moisture content and the wet-to-dry ratio were calculated. The lung moisture content = [(lung wet weight−lung dry weight)/lung wet weight] × 100%, and the wet-to-dry ratio = lung wet weight/lung dry weight.

### Spirometry

Mice were placed in a FinePointe WBP volume tracing chamber (Data Sciences International, USA) and allowed to acclimate for 5 min in a controlled environment. After respiratory waveforms stabilized, data were recorded for 5 min. The respiratory rate (f), minute volume (MV), tidal volume (TV), peak inspiratory flow (PIF), and peak expiratory flow (PEF) were quantified for each group.

### Biochemical indices

The levels of glutathione peroxidase (GSH-Px, A005-1–2, Nanjing Jiancheng Bioengineering Institute), total superoxide dismutase (T-SOD, A001-1-1, Nanjing Jiancheng Bioengineering Institute), and malondialdehyde (MDA, A003-1-1, Nanjing Jiancheng Bioengineering Institute) in seurm were measured using specific kits following the manufacturers’ protocols.

### Enzyme-linked immunosorbent assay (ELISA) analysis

The levels of IL-6 (YJ098430, Shanghai Enzyme-linked Biotechnology Co., Ltd.), IL-1β (YJ301814, Shanghai Enzyme-linked Biotechnology Co., Ltd.) and TNF-α (YJ002095, Shanghai Enzyme-linked Biotechnology Co., Ltd.) in bronchoalveolar lavage fluid (BALF), cell supernatant and lung organoids supernatant were detected according to the instructions.

### The levels of reactive oxygen species (ROS) and apoptosis

Lung: The lungs were perfused with phosphate-buffered saline (PBS) and digested with pancreatic enzyme. The cells were then pelleted at 1200 rpm for 5 min at 4 °C. The levels of ROS were measured by treating cells with 1 mL of DCFH-DA diluent (10 μmol/L, CA1410, Solarbio Life Science, China). And the cell apoptosis levels were detected using an Annexin V-PE/7-AAD Apoptosis Detection Kit (559763, BD PharmingenTM). The 5 μL of 7-AAD and 5 μL of PE probes were added to each tube.

Cells: After the completion of drug administration, the cells were digested with trypsin, transferred to flow cytometry tubes, centrifuged, and the supernatant was discarded. Subsequently, the cell processing and incubation procedures were the same as those for lung tissue.

### Flow cytometry analysis of the level of immune cells in the blood and lung

Mouse blood and lung cells were aliquoted into five flow cytometry tubes, each containing 100 μL of cells. Macrophages and neutrophils were identified using anti-CD45 (11–0041-82, eBioscience, USA), anti-Ly6G (11966882, Thermo Fisher Scientific, China), and anti-F4/80 (48480180, Thermo Fisher Scientific, China) antibodies. The cellular fluorescence intensity was analyzed by flow cytometry.

### UPLC-Q-TOF-MS analysis

The methods for processing the serum and lung tissues, as well as the data processing methods, were all carried out in accordance with the methods used in our previous research. In addition, equal volumes of each sample were pooled to prepare quality control (QC) samples, which were used to monitor the stability and reproducibility of the analytical system. All samples were analyzed using ultra-high-performance liquid chromatography coupled with quadrupole time-of-flight mass spectrometry (UPLC-Q-TOF-MS). Mass spectrometry conditions: A Bruker MaXis HD Q-TOF mass spectrometer was employed with a scan range of m/z 50–1000 and a collision energy of 8.0 eV. The capillary voltage was set to 3.5 kV in positive ion mode (ESI⁺) and 3.2 kV in negative ion mode (ESI⁻). Chromatographic conditions: Separation was performed on a Thermo Acclaim^™^ RSCL 120 C18 column (2.1 mm × 100 mm, 2.2 μm). The column temperature was maintained at 40 °C, with a flow rate of 0.3 mL/min and an injection volume of 2 μL. The mobile phases consisted of 0.1% formic acid in water (mobile phase A) and acetonitrile (mobile phase C). However, some parts of the methods in this study were quite different from those in previous ones. In the mobile phase elution procedure of lung tissue, the following settings are adopted: 0–5 min, 10–65% C; 5–6 min, 65–70% C; 6–16 min, 70–80% C; and 16–19 min, 80–95% C. After data acquisition by UPLC-Q/TOF-MS, the raw data were imported into Profile Analysis for preprocessing, including filtering, normalization, and calibration, to generate text files (txt). The obtained positive and negative mode.txt files were then imported into SIMCA 14.0 software for multivariate statistical analysis. Principal component analysis (PCA) and orthogonal partial least squares discriminant analysis (OPLS-DA) were performed to generate PCA and OPLS-DA score plots, as well as variable importance in projection (VIP) lists. A 200-permutation test was subsequently conducted to validate the robustness of the OPLS-DA model. Potential biomarkers were screened based on the criteria of *P* < 0.05 and VIP > 3. The accurate molecular weights of candidate metabolites were determined using DataAnalysis 4.4 software. Further identification of these metabolites was performed by searching databases including the Human Metabolome Database (HMDB, http://www.hmdb.ca) and the Kyoto Encyclopedia of Genes and Genomes (KEGG, https://www.kegg.jp). Peak areas of each biomarker were obtained using Quant Analysis software for semi-quantitative analysis. Finally, metabolic pathway enrichment analysis was performed using MetaboAnalyst (https://www.metaboanalyst.ca/home.xhtml) and KEGG databases.

### Data acquisition

The GSE40885 data sets (Human alveolar macrophages were collected by bronchoscope lavage and stimulated by lipopolysaccharide to analyze the gene expression profile) in GEO database were used to analyze the genes expression in alveolar macrophages in BALF. RMA algorithm has been used for background correction and normalization in the original research of GSE40885 data set. According to *P* < 0.05 and log^2^FC > 1, differential genes were screened. And perform KEGG analysis on the top 30 differentially expressed genes. The cibersort algorithm was used to analyze the gene expression matrix. The abundance of each immune cell in the tissue was estimated based on the gene expression levels of marker genes, and the abundance of each cell was subjected to the wilcox test to calculate the abundance of immune cells between the two groups.

### Molecular docking

Initially, ChemDraw was employed to convert the small molecule ligands AAD, into SDF files. The PDB file of IL-17A (8DYG), IL-17E (7UWJ), IL-17F (6HGO) was obtained from the RCSB PDB database. The protein structure of IL-17C was predicted based on the UniProt accession number Q9P0M4 using the AlphaFold3 model (Figure S2). Using the SailVina platform, receptors, ligands, and docking sites were prepared, followed by molecular docking. Docking scores were extracted, and results were analyzed. Finally, the binding sites were visualized and analyzed.

### qPCR

Total RNA was extracted from the lung tissues of each group of mice (R1200, Beijing Solab Technology Co., Ltd.) and from the zebrafish (G3684-50T, Ltd.Wuhan Seville Biotechnology Co., Ltd.) using different kits, and which was reverse transcribed into cDNA using the SweScript RT Ⅰ kit. cDNA was amplified with 2 × Universal Blue SYBR Green qPCR Master Mix, and mRNA expression was quantified using the 2-^ΔΔ^Ct method (Tables 1, 2). Primer sequences are listed below:

**Table 1:** Primer sequence

Gene (Mouse)	Primer sequence (5'−3')
IL-17A	Sense: CTGTGTCTCTGATGCTGTTGCTG
	Anti-sense: CGTGGAACGGTTGAGGTAGTC
IL-17E	Sense: CGGCATGTACCAGGCTGTTG
	Anti-sense: AGCTCCACTTCAGCCACTCCT
IL-17F	Sense: AGACAGCACCATGAACTCCG
	Anti-sense: ACAATGGGCTTGACACAGGT
IL-17C	Sense: GAAACCCCGAAGCCATAGGA
	Anti-sense: GTGGGTAGCGGTTCTCATCTGT
GAPDH	Sense: CCTCGTCCCGTAGACAAAATG
	Anti-sense: TGAGGTCAATGAAGGGGTCGT

**Table 2:** Primer sequence

Gene (Zebrafish)	Primer sequence (5'−3')
IL-17A	Sense: CAATCCCATCCATCCAATCAA
	Anti-sense: GGACCTCAACGCCGTCTATCA
IL-1β	Sense: GTCACACTGAGAGCCGGAAG
	Anti-sense: GCAGGCCAGGTACAGGTTAC
IL-6	Sense: GTCCCCGTGTTCAGCAGTATG
	Anti-sense: GGCAGCGGTCTGAAGGTTT
TNF-α	Sense: AGTCGGGTGTATGGAGGGTGTT
	Anti-sense: TGATTGCCCTGGGTCTTATGG
GAPDH	Sense: AGAAACCTGCCAAGTATGATGAG
	Anti-sense: TTGCTGTAACCGAACTCATTGTC

**Table 3:** ^1^H-NMR and ^13^C-NMR data of the compound AAD (in CD_3_OD, *δ* in ppm)

No	*δ*_H_	*δ*_C_	No	*δ*_H_	*δ*_C_
1	2.23 (2H, m)	35.3	18	1.07 (3H, s)	27.1
2	2.80 (1H, m) 2.05 (1H, m)	35.1	19	1.16 (3H, s)	26.0
3		223.6	20	2.80 (1H, m)	29.9
4		48.1	21	0.98 (3H, d, *J* = 7.0 Hz)	24.3
5	2.18 (1H, m)	49.7	22	1.56 (1H, m) 1.28 (1H, m)	42.0
6	1.49 (1H, m) 1.30 (1H, m)	21.2	23	5.03 (1H, m)	71.3
7	2.29 (1H, m) 2.08 (1H, m)	32.0	24	4.71 (1H, d, *J* = 4.4 Hz)	80.1
8		42.0	25		72.5
9	1.77 (1H, d, *J* = 10.9 Hz)	50.7	26	1.03 (3H, overlap)	20.8
10		38.2	27	1.02 (3H, overlap)	21.1
11	3.75 (1H, m)	70.6	28	1.17 (3H, s)	30.8
12	2.63 (1H, m) 2.04 (1H, m)	34.7	29	1.03 (3H, overlap)	26.0
13		139.6	30	1.18 (3H, s)	24.3
14		58.3	31		172.2
15	1.95 (1H, m) 1.32 (1H, m)	31.8	32	2.08 (3H, s)	20.5
16	2.28 (1H, m) 1.99 (1H, m)	30.4	33		171.9
17		135.5	34	2.01 (3H, s)	20.4

### Western blot

The protein extraction and incubation conditions were as described previously. The primary antibodies: IL-17A (1:2000, Proteintech, 26163-1-AP), p-NF-κB-P65 (1:5000, Proteintech, 82335-1-RR), NF-κB-P65 (1:1000, Proteintech, 10745-1-AP) and β-actin (1:25000, Abclonal, AC026). After incubation with goat anti-rabbit IgG secondary antibody (1:20000, LI-COR, 926–32211) for 1 h, protein bands were visualized using the Odyssey^®^ CLx Imaging System and analyzed with Image Studio Ver 5.2.

### Immunofluorescence

Lung tissue sections and lung organoids were deparaffinized, antigen-repaired, and blocked with serum. Primary antibodies (IL-17A, 1:100; p-NF-κB-P65, 1:1000) were applied and incubated at 4 °C overnight. The secondary antibody was added and incubated at 37 °C for 30 min. Then stained with DAPI for 10 min. Finally, the sections were blocked, and fluorescence expression was analyzed using a digital slide scanner.

### Cellular immunofluorescence

After the cell culture is completed, the procedures described in the literature are followed. The primary antibodies used are IL-17A (1:100), p-NF-κB-P65 (1:1000), NF-κB-P65 (1:500), and β-actin (1:200). Images are collected and fluorescence intensity is analyzed using the Operetta high-content imaging system.

### Zebrafish behavioral experiments

Behavioral monitoring was conducted using a 24-well plate with one fish per well. The monitoring software was launched, and the 'video source' was selected in high-definition camera mode. A circular area was then defined, with the first well containing juvenile fish circled. The multi-well plate parameters were selected to generate the 24-well plate, followed by the setting of the scale length. After completing all settings, the experiment was initiated to record the zebrafish's movement trajectory within 3 min for subsequent experimental analysis.

### Data analysis

Data were analyzed using SPSS 20.0 statistical software. For multi-group comparisons, one-way ANOVA was employed. When homogeneity of variances was assumed (*P* > 0.05), pairwise comparisons were conducted using the LSD test; when homogeneity of variances was not assumed (*P* < 0.05), the Tamhane's T2 test was used. Data analysis results were presented as mean ± standard deviation. Data were presented as mean ± standard deviation. *P* < 0.05 indicated significant differences, while a *P* < 0.01 indicated highly significant differences.

## Results

### Structural identification of compound AAD

**Fig. 1:**
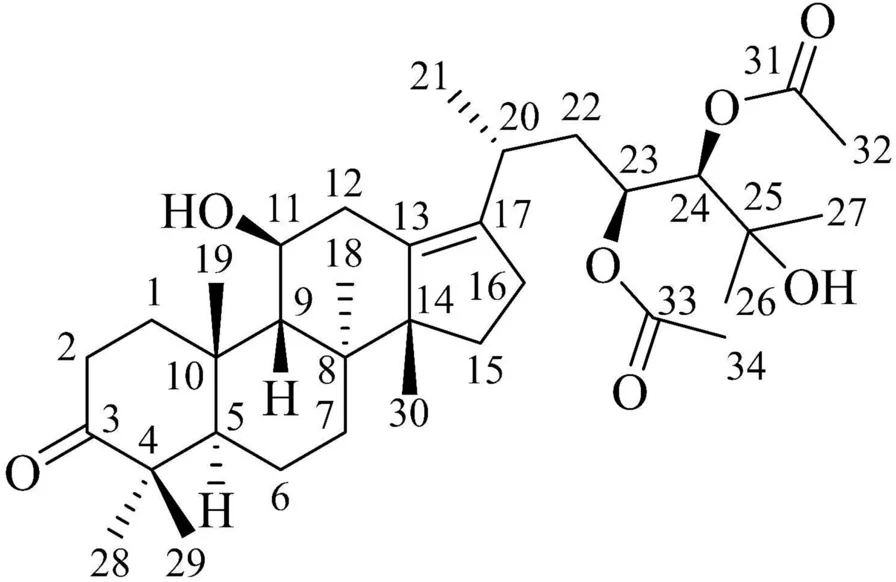
The structural formula of the compound AAD

The ^1^H-NMR spectrum (Table 3) of compound AAD reveals ten methyl signals in the high-field region [*δ*_H_ 2.08 (3H, s, H-32), 2.01 (3H, s, H-34), 1.18 (3H, s, H-30), 1.17 (3H, s, H-28), 1.16 (3H, s, H-19), 1.07 (3H, s, H-18), 1.03 (3H, overlap, H-29), 1.02 (3H, overlap, H-27), 1.03 (3H, overlap, H-26), 0.98 (3H, d, J = 7.0 Hz, H-21)], with *δ*_H_ 2.08 observed at lower fields, suggesting its direct linkage to a carbonyl group. Additionally, three oxygen-hydrogen signals are detectable in the low-field region [*δ*_H_ 5.03 (1H, m, H-23), 4.71 (1H, s, H-24), 3.75 (1H, m, H-11)]. Furthermore, *δ*_H_ 1.77 (1H, d, *J* = 10.8 Hz) is the characteristic signal of the prototerpene-type triterpene H-9. In the ^13^C-NMR spectrum (Table 3), a carbonyl group [δ_C_ 223.6 (C-3)] and a set of double bonds [*δ*_C_ 139.6 (C-13), 136.3 (C-17)] are observed in the low-field region. The combination of *δ*_C_ 172.2, 171.9 and *δ*_H_ 2.08, 2.01 indicates that AAD contains two acetyl group. Additionally, the characteristic carbon signals at δ_C_ 70.6, 71.3, 80.1, and 72.5 correspond to the C-11, C-23, C-24, and C-25 positions of the prototerpene-type triterpenes. Based on the two-dimensional spectrum and known literature (Fig. 1), the compound is identified as alisol A 23,24-diacetate (AAD).

### AAD safety evaluation

**Fig. 2:**
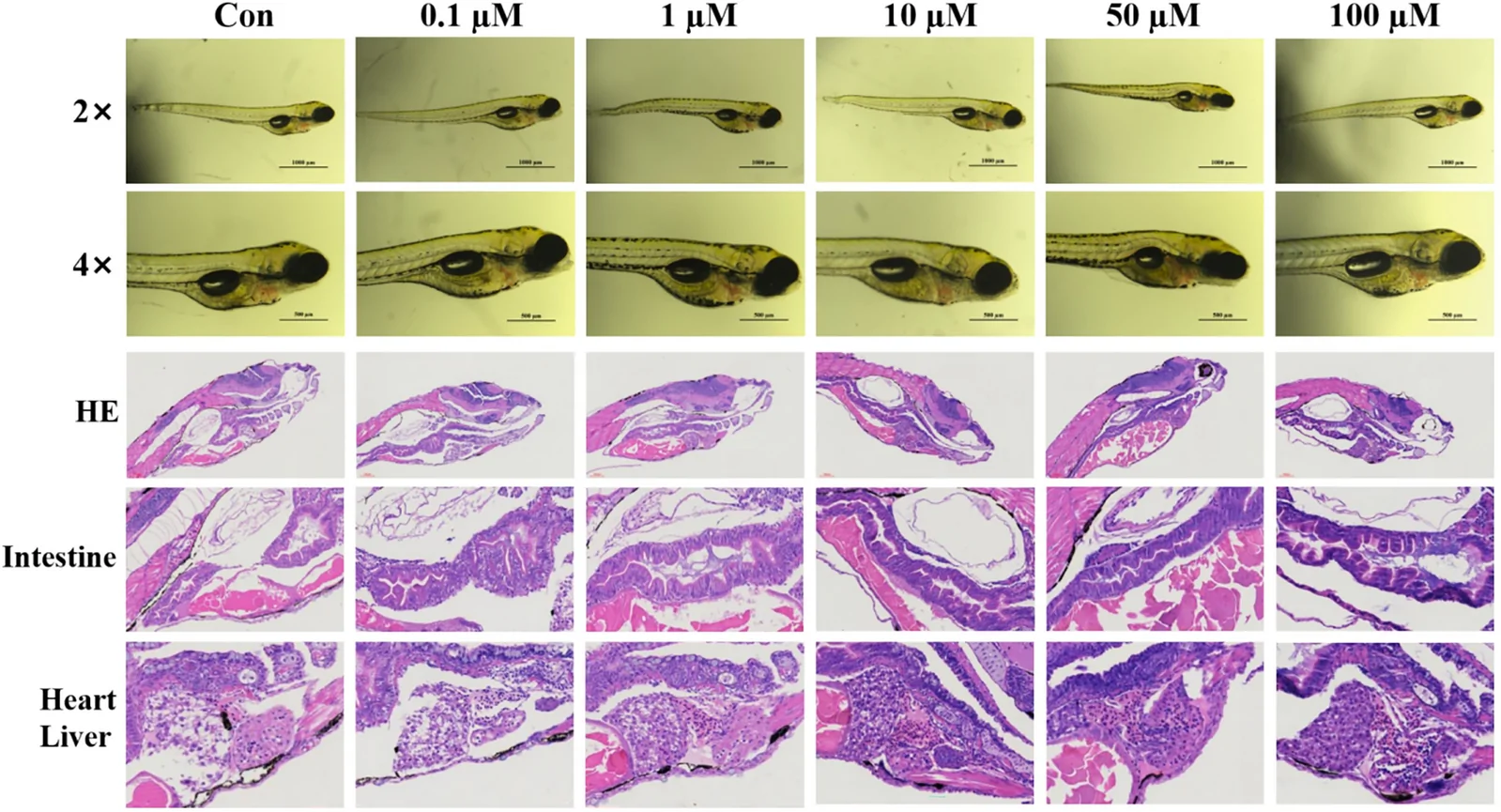
The effects of AAD on zebrafish

First, we evaluated the safety of AAD. We exposed zebrafish to different concentrations of AAD and observed the results. The results are shown in Fig. 2. The body colors of each group of zebrafish were bright and uniform, without any abnormal manifestations such as darkened body color or local pigment loss. The body shape was relaxed, the ratio of the trunk to the tail was harmonious, and there were no deformities such as spinal curvature, shortened body axis, pericardial edema, or yolk sac edema. The morphology of these zebrafish was consistent with that of the blank control group.

### AAD significantly ameliorates lung injury in mice with ALI

**Fig. 3:**
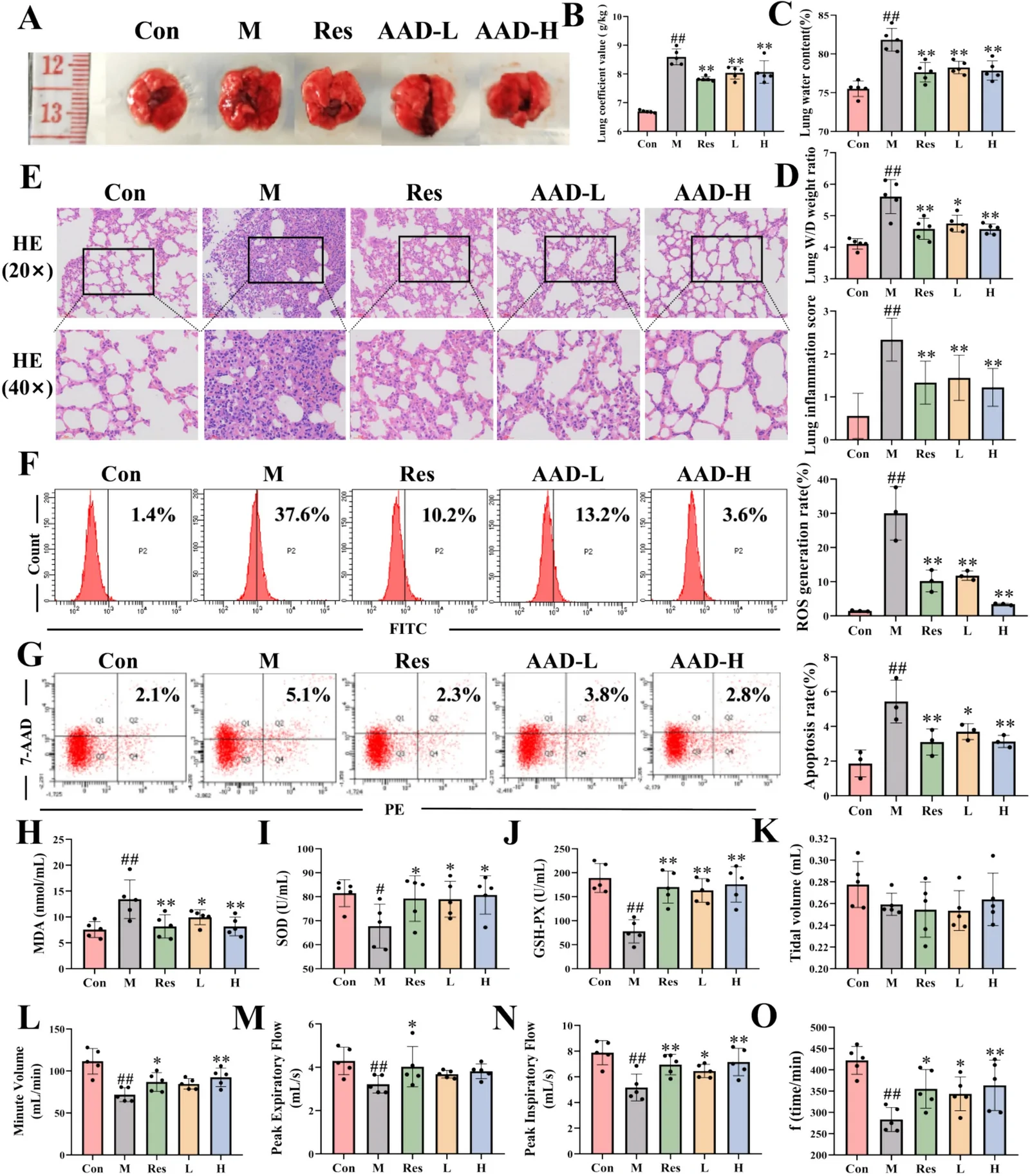
The effect of AAD on pulmonary function in mice with ALI. **A** The lung dissection images. **B**–**D** The lung coefficient, lung water content, and lung wet-to-dry weight ratio in each group of mice (n = 5). **E** HE staining and inflammation quantification in each group of mice (n = 3). **F**, **G** Representative images and quantitative results of ROS and apoptosis levels detected in mouse lung tissue by flow cytometry (n = 3). **H**–**J** The serum levels of MDA, SOD, and GSH-Px in each group of mice (n = 5). **K**–**O** Lung function-related indicators (TV, MV, PEF, PIF, f) in each group of mice (n = 5). Con means the Control group; M means the model group; Res means the Resveratrol group; AAD-L (**L**), AAD-H (**H**) means the low and high dose of alisol A 23,24-diacetate group, respectively. The data were expressed as the mean ± SD. ^#^*P* < 0.05, ^##^*P* < 0.01 compared with the Con group. ^*^P < 0.05, ^**^P < 0.01 compared with the M group

LPS can induce pulmonary edema and structural damage in mouse lung tissue, leading to increased apoptosis and elevated levels of oxidative stress, thereby resulting in decreased pulmonary function [22, 23]. Our experimental results demonstrated that the M group mice exhibited significant pulmonary edema, with markedly increased lung water content and wet-to-dry weight ratio (Fig. 3A–C, *P* < 0.01). HE staining revealed evident inflammatory infiltration in the lung tissue of the model group mice, along with significant thickening of the alveolar septa (Fig. 3D, E, *P* < 0.05 or *P* < 0.01). Following pharmacological intervention, AAD significantly improved pulmonary edema and alleviated lung injury (*P* < 0.05 or *P* < 0.01). Flow cytometry and kit assay results indicated that apoptosis and oxidative stress levels were significantly elevated in the model group mice, whereas AAD significantly reduced both apoptosis and oxidative stress levels (Fig. 3F–J, *P* < 0.05 or *P* < 0.01). Further pulmonary function tests revealed that AAD significantly increased MV, PEF, PIF, and f (Fig. 3K–O, *P* < 0.05 or *P* < 0.01). These results collectively demonstrate that AAD can significantly ameliorate lung injury and pulmonary function in ALI mice.

### AAD significantly improves metabolic dysregulation in the lung tissue of ALI mice

**Fig. 4:**
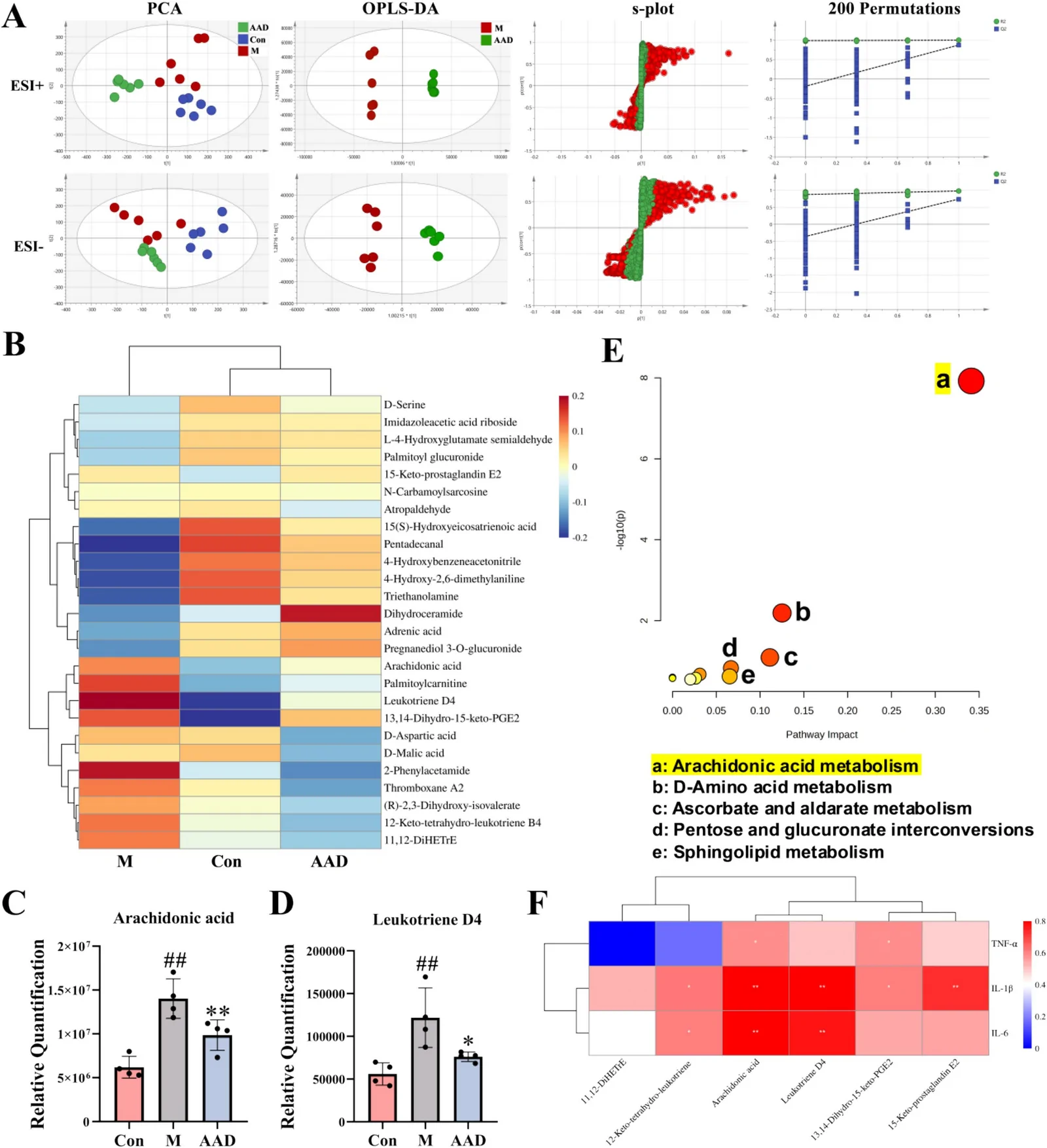
The effect of AAD on metabolic levels in lung tissues of ALI mice. **A** PCA score plots, OPLS-DA scatter plots, S-plot scatter plots, and 200 permutation test plots for each group of lung tissues in the positive and negative ion modes. **B** Heatmap of cluster analysis for potential biomarkers in lung tissue. **C**, **D** Quantitative expression of metabolites related to the arachidonic acid metabolic pathway (n = 4). **E** Enrichment analysis of biomarker metabolic pathways. **F** Correlation analysis between metabolites associated with arachidonic acid metabolism and inflammatory factors. The data were expressed as the mean ± SD. ^#^*P* < 0.05, ^##^*P* < 0.01 compared with the Con group. ^*^P < 0.05, ^**^P < 0.01 compared with the M group

The tracheal instillation model enables direct targeting of LPS to lung tissue, inducing metabolic disturbances that are subsequently exacerbated by blood circulation, thereby further aggravating pulmonary injury. Consequently, we first assessed metabolic levels in lung tissue. PCA revealed distinct clustering of samples across different groups under both positive and negative ion modes (Fig. 4A). Further OPLS-DA analysis identified significant metabolic characteristic differences between the AAD group and the M group, indicating that AAD markedly modulated metabolic disturbances in LPS-induced ALI mouse lung tissues (Fig. 4A). Using VIP > 3 and *P* < 0.05 as screening criteria, biomarkers were screened, quantified, and clustered. The results, as shown in the Fig. 4B, demonstrated that the AAD group clustered first with the normal group, suggesting that post-administration, the abnormal metabolism in ALI mice was restored. Further metabolic pathway enrichment analysis revealed that arachidonic acid metabolism was the primary pathway disrupted (Fig. 4C–E). Correlation analysis indicated a significant association between arachidonic acid-related metabolites and inflammatory cytokine markers (Fig. 4F).

### GEO database differential gene and enrichment analysis

**Fig. 5:**
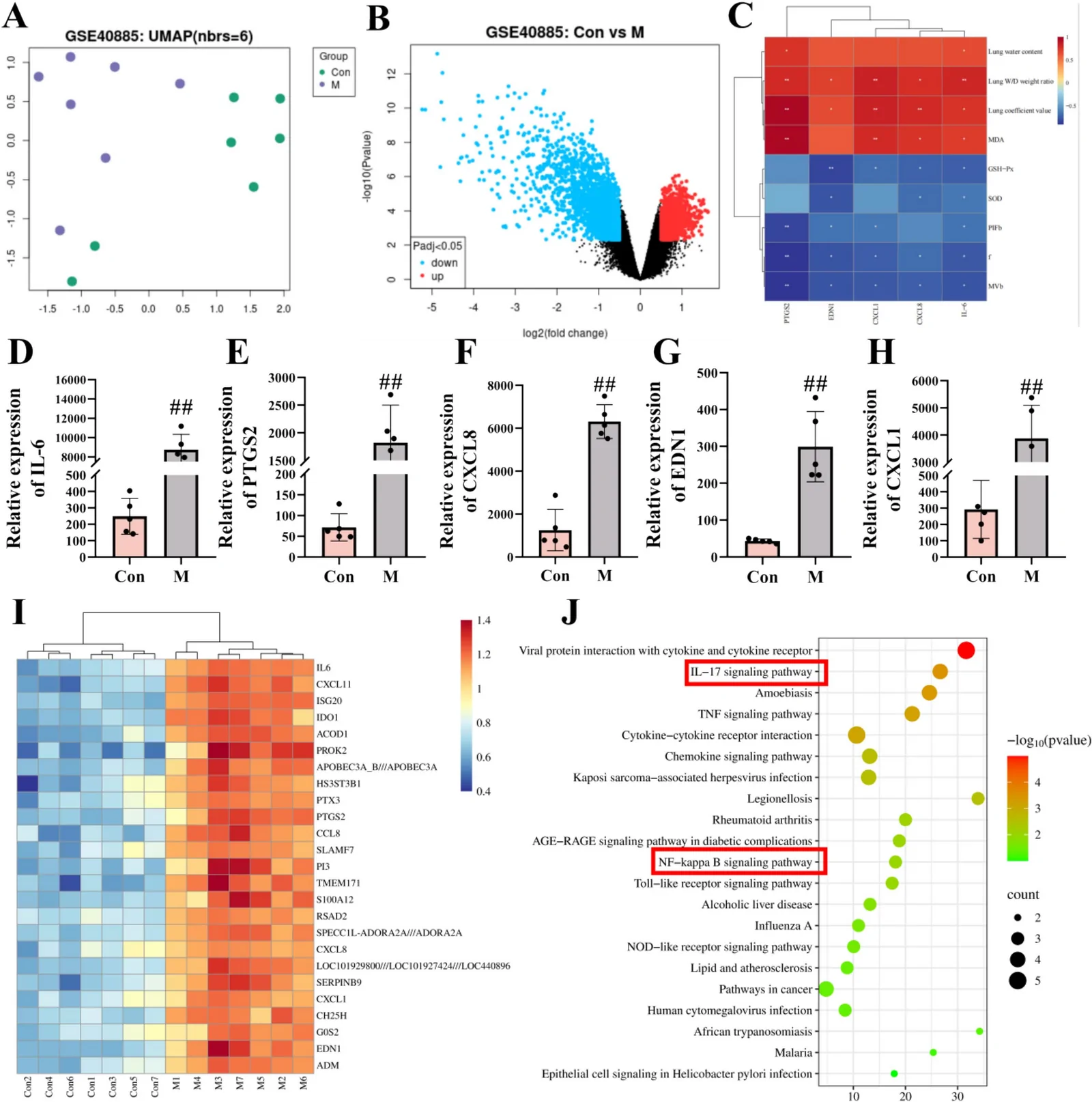
The IL-17/NF-κB signaling pathway plays a significant role in ALI. **A** The UMAP diagram. **B** The volcano map. **C**, **D** Correlation analysis of genes with lung injury and lung function indicators. **D**–**H** Expression levels of genes related to the IL-17 and NF-κB signaling pathways (n = 5). **I** Heat map analysis of the top 30 genes. **J** KEGG enrichment analysis of the top 30 genes. The data were expressed as the mean ± SD. ^#^*P* < 0.05, ^##^*P* < 0.01 compared with the Con group

Download the GSE40885 dataset from the GEO database, which includes lung tissue samples from 7 samples of BALF from the lung tissues of healthy individuals and 7 samples of BALF from human lung tissues after intratracheal injection of LPS for 6 h. As shown in Fig. 5A, the UMAP shows that the distance between the two groups is far away and there is no obvious intersection. The expression heat map of the 30 genes with significant differences, sorted by logFC value, is shown in Fig. 5I. KEGG enrichment analysis of these genes revealed that the IL-17 signaling pathway ranked the highest (Fig. 5J). The IL-17 signaling pathway can directly activate the NF-κB pathway and enhance the release of pro-inflammatory factors. By intersecting and quantitatively analyzing the genes enriched in both pathways, it was found that genes such as IL-6 and PTGS2 were significantly elevated in ALI patients (Fig. 5D–H, *P* < 0.05 or *P* < 0.01). Correlation analysis of these genes with lung injury-related indicators revealed a significant positive correlation with lung water content, wet-to-dry ratio, and MDA levels (Fig. 5C). These results suggest that the IL-17 and NF-κB pathway plays an important role in ALI.

### AAD ameliorates lung injury in ALI mice through IL-17A/NF-κB signaling pathway

**Fig. 6:**
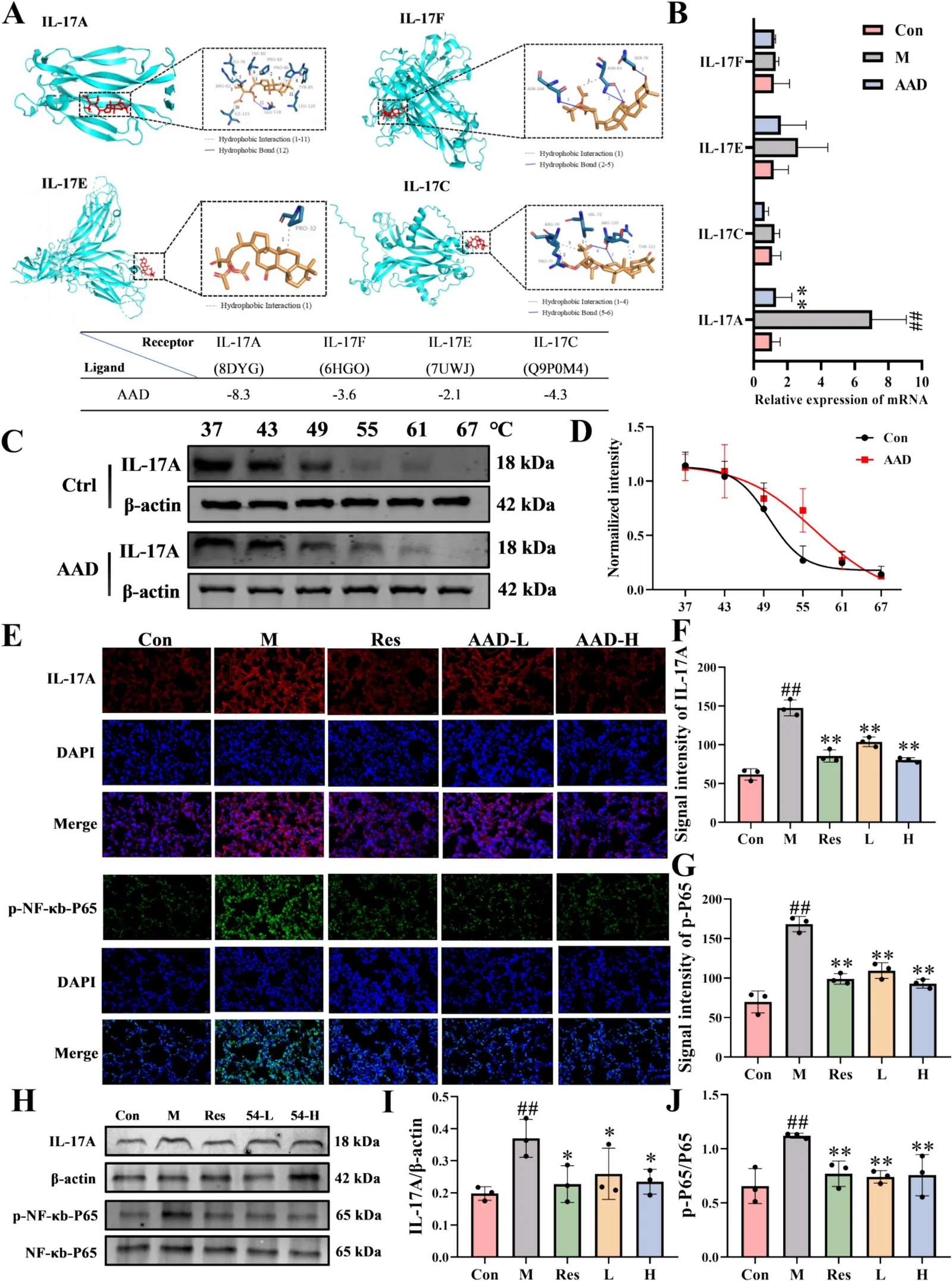
The effect of AAD on the IL-17A/NF-κB signaling pathway. **A** Molecular docking results of IL-17A, IL-17E, IL-17F, and IL-17C with AAD. **B** Detection of the levels of IL-17A, IL-17E, IL-17F, and IL-17C in the lung tissues of each group of mice using PCR method. **C**, **D** CETSA detected the effect of AAD on the thermal stability of IL-17A protein. **E**–**G** Detection of the expression levels of proteins related to the IL-17A/NF-κB signaling pathway in the lung tissues of each group of mice by immunofluorescence method. **H**–**J** Detection of the expression levels of proteins related to the IL-17A/NF-κB signaling pathway in the lung tissues of each group of mice by WB method. The data were expressed as the mean ± SD, n = 3. ^#^*P* < 0.05, ^##^*P* < 0.01 compared with the Con group. ^*^P < 0.05, ^**^P < 0.01 compared with the M group

In order to further analyze the target points through which AAD exerts its effects, we conducted molecular docking and PCR tests on the subtypes of the IL-17 family. The results showed that AAD had the highest docking score with IL-17A, and the changes were most significant in the mice (Fig. 6A, B, *P* < 0.01). The results of CETSA further showed that AAD can improve the stability of IL-17A protein, and there may be a direct combination between them (Fig. 6C, D). Further WB and immunofluorescence results indicated that the levels of IL-17A and p-NF-κB-P65 in the lung tissues of the model group mice were significantly increased (Fig. 6E–J, *P* < 0.05 or *P* < 0.01). After administering AAD intervention, different doses of AAD could significantly reduce the protein expression levels (*P* < 0.05 or *P* < 0.01). These results suggest that AAD may improve LPS-induced acute lung injury through the IL-17A/NF-κB signaling pathway.

### AAD significantly improves immune disorders in ALI mice

**Fig. 7:**
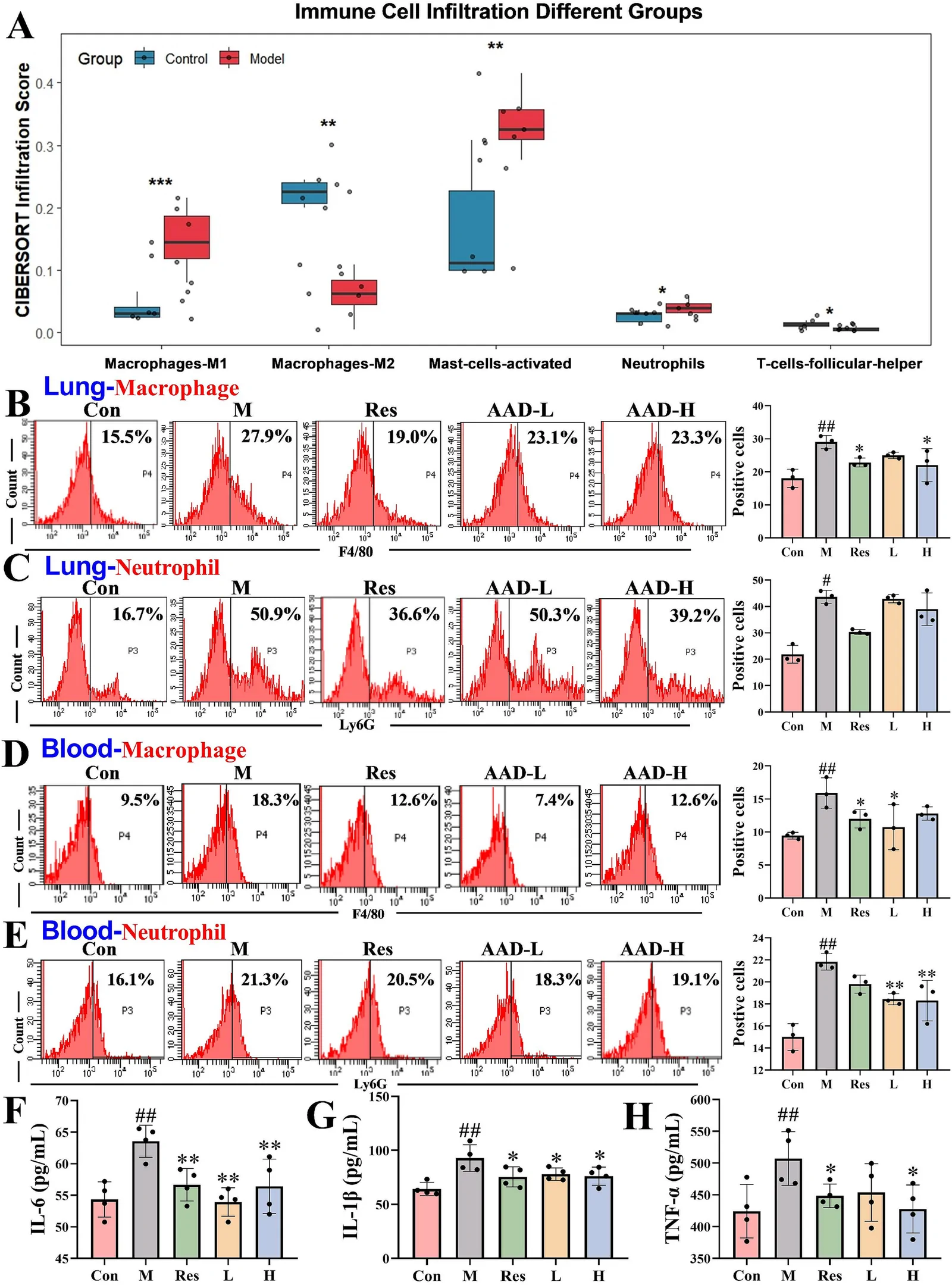
The effect of AAD on immune inflammation in ALI mice. **A** The immune infiltration analysis results of the GSE40885 database. **B**, **C** Flow cytometry representative images and quantitative results of macrophages and neutrophils in lung tissue (n = 3). **D**, **E** Flow cytometry representative images and quantitative results of macrophages and neutrophils in peripheral blood (n = 3). **F**–**H** Levels of inflammatory factors in serum (n = 4). The data were expressed as the mean ± SD. ^#^*P* < 0.05, ^##^*P* < 0.01 compared with the Con group. ^*^P < 0.05, ^**^P < 0.01 compared with the M group

The IL-17A/NF-κB axis can activate macrophages and neutrophils, and macrophages can promote the release of IL-6, IL-1β, and TNF-α, while neutrophils can further amplify their effects [24, 25]. The immune infiltration analysis of the GSE40885 database showed that the M1 type macrophages and neutrophils in the model group were significantly increased (Fig. 7A). The results of flow cytometry showed that the levels of macrophages and neutrophils in the lung tissue and blood of ALI mice were significantly increased (Fig. 7B–E, *P* < 0.05 or *P* < 0.01). The release of inflammatory factors IL-6, IL-1β, and TNF-α was also increased (Fig. 7F-H, *P* < 0.05 or *P* < 0.01). After drug intervention, AAD significantly restored the disorder of immune cells and reduced the levels of inflammatory factors (*P* < 0.05 or *P* < 0.01). These results indicate that AAD can improve the immune disorder of ALI mice through IL-17A/NF-κB signal pathway.

### AAD significantly ameliorates LPS-induced lung organoid injury

**Fig. 8:**
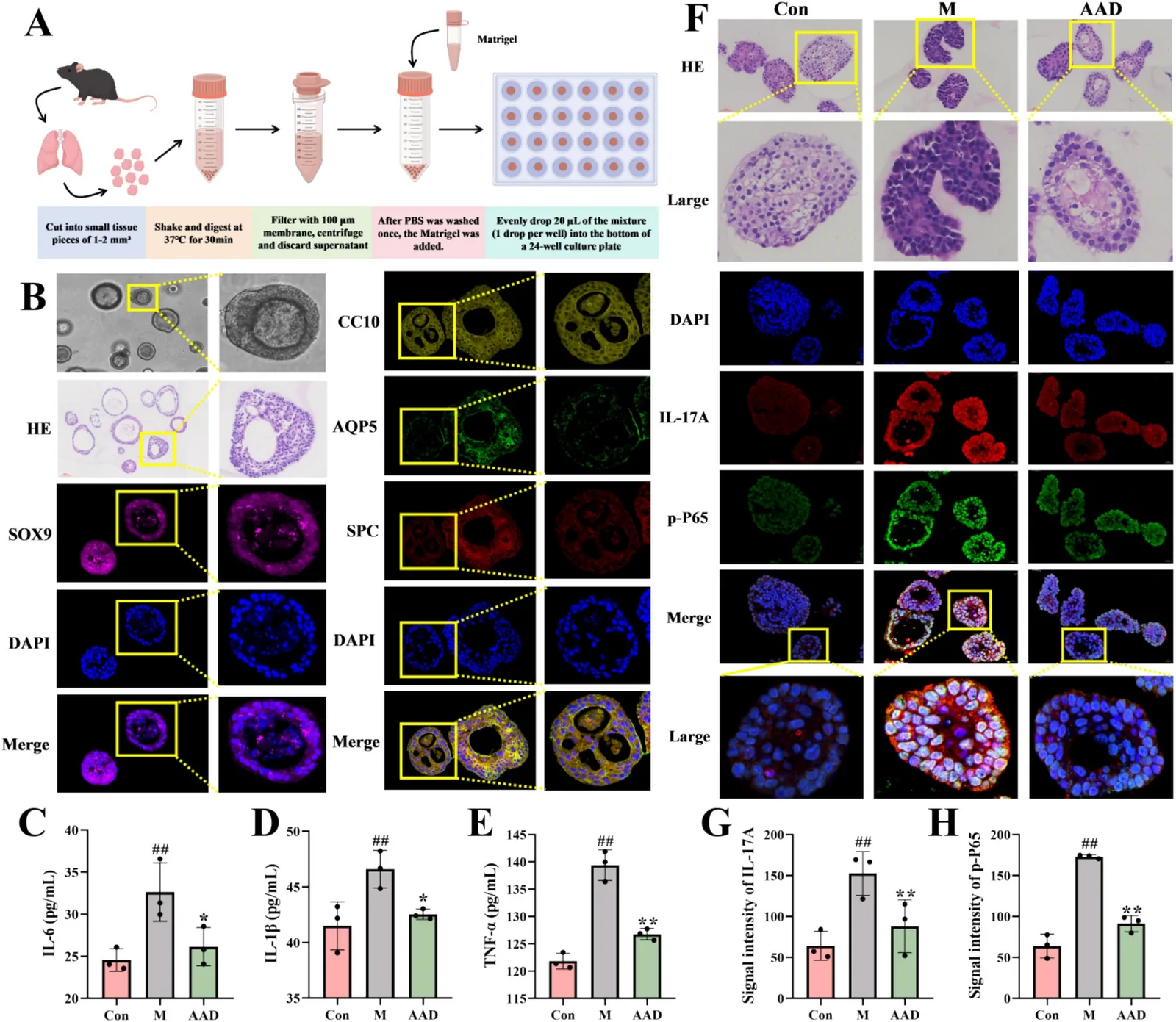
The effect of AAD on LPS-induced lung organoid injury. **A** Lung organoid experimental flowchart. **B** Identification of pulmonary organoids. **C**–**E** The level of inflammatory factors in the supernatant. **F**–**H** HE staining, immunofluorescence staining and their quantitative results. The data were expressed as the mean ± SD. ^#^*P* < 0.05, ^##^*P* < 0.01 compared with the Con group. ^*^P < 0.05, ^**^P < 0.01 compared with the M group. The data were expressed as the mean ± SD, n = 3. ^#^*P* < 0.05, ^##^*P* < 0.01 compared with the Con group. ^*^P < 0.05, ^**^P < 0.01 compared with the M group

Lung organoids, as three-dimensional in vitro models, can highly simulate the structure, cellular heterogeneity and microenvironmental interactions of in vivo lung tissues [26]. Therefore, we constructed a lung organoid model (Fig. 8A, B). After LPS modeling, the HE staining results showed that the cell nuclei were significantly shrunken and the alveolar structure was incomplete (Fig. 8F). After drug intervention, the phenomenon of nuclear shrinkage was significantly improved, and the alveolar structure was partially restored. The immunofluorescence results showed that the protein levels of IL-17A and p-NF-κB-P65 in the model group organoids were significantly increased, and after drug intervention, the protein expression was significantly reduced (Fig. 8F–H, *P* < 0.05 or *P* < 0.01). Further detection of the levels of inflammatory factors in the supernatant showed that AAD significantly reduced the levels of IL-6, IL-1β and TNF-α (Fig. 8C–E, *P* < 0.05 or *P* < 0.01). These results indicate that AAD can significantly improve the lung organoid damage induced by LPS.

### AAD ameliorates LPS-induced Beas-2B cell injury through IL-17A/NF-κB

**Fig. 9:**
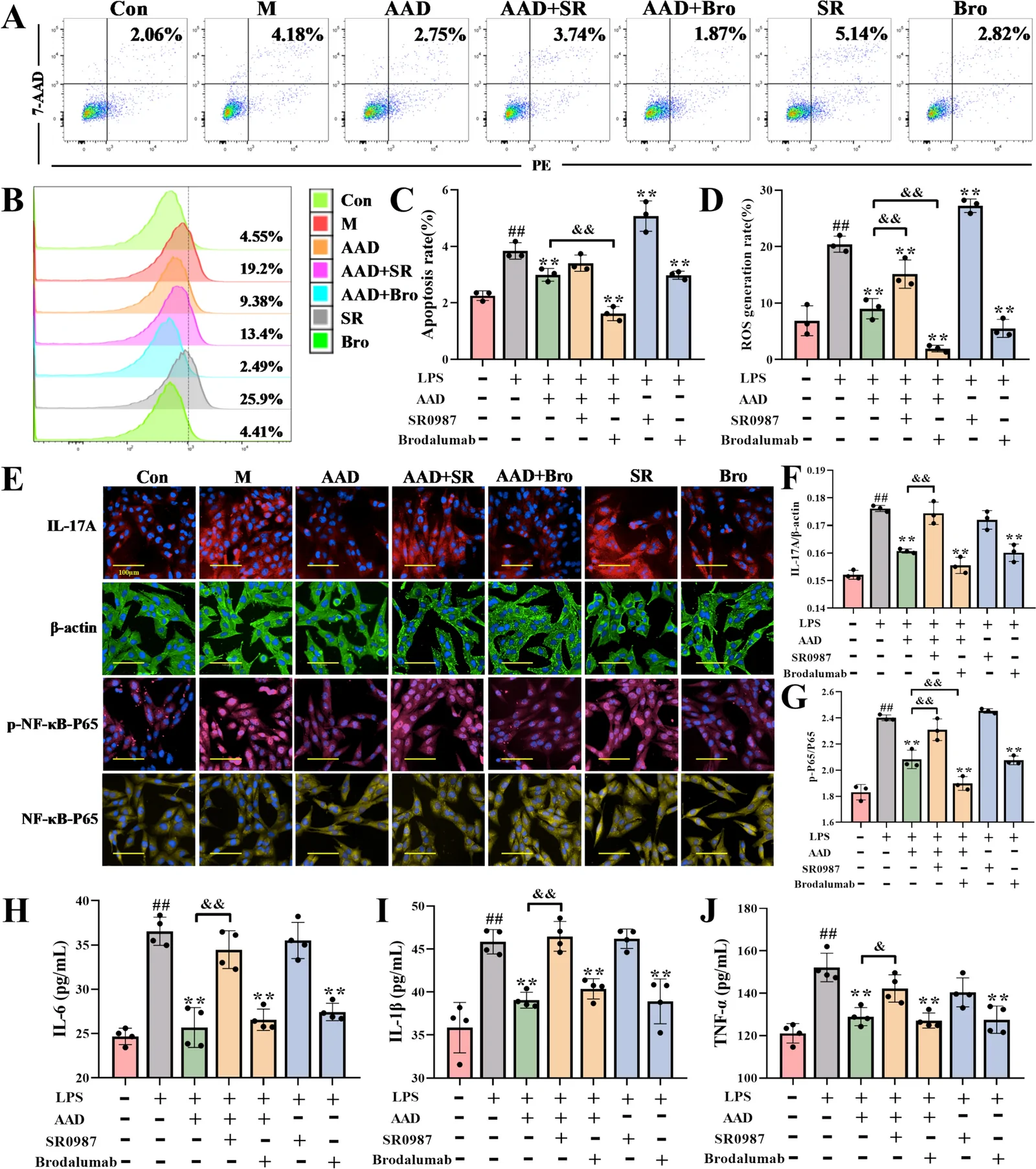
The effect of AAD on LPS-induced injury in Beas-2B cells. **A**–**D** Flow cytometry representative images and quantitative results of apoptosis and ROS levels in Beas-2B cells (n = 3). **E**–**G** Representative images and quantitative results of related proteins in IL-17A/NF-κB in Beas-2B cells (n = 3). **H**–**J**. The levels of inflammatory factors in the supernatant of Beas-2B cells (n = 4). SR0987: a RORγt agonist (pro-Th17/IL-17 signaling). Brodalumab:an anti-IL-17RA monoclonal antibody (blocks IL-17 receptor signaling). The data were expressed as the mean ± SD. ^#^*P* < 0.05, ^##^*P* < 0.01 compared with the Con group. ^*^P < 0.05, ^**^P < 0.01 compared with the M group. ^$^*P* < 0.05, ^$$^*P* < 0.01 compared with the AAD group

To verify whether AAD improves lung injury through the IL-17A/NF-κB preference pathway, we established a cell model and combined the agonist and inhibitor of IL-17. The results showed that the levels of apoptosis and ROS in the model group cells were significantly increased (Fig. 9A–D, *P* < 0.01), the expression of IL-17A and p-NF-κB-P65 proteins was enhanced (Fig. 9E–G, *P* < 0.01), and a large amount of inflammatory factors IL-6, IL-1β, and TNF-α were released (Fig. 9H–J, *P* < 0.01). After AAD and IL-17 antagonists intervention, the levels of ROS and apoptosis were significantly inhibited, the levels of IL-17A and p-NF-κB-P65 proteins and the release of inflammatory factors were reduced (*P* < 0.01). However, the above effects were reversed when the IL-17 agonist was added, and the improvement effect was further enhanced when the IL-17 antagonist was added (*P* < 0.05 or *P* < 0.01). These results indicate that AAD improves LPS-induced Beas-2B cell injury through the IL-17A/NF-κB pathway.

### AAD improves LPS-induced damage in zebrafish through IL-17A target

**Fig. 10:**
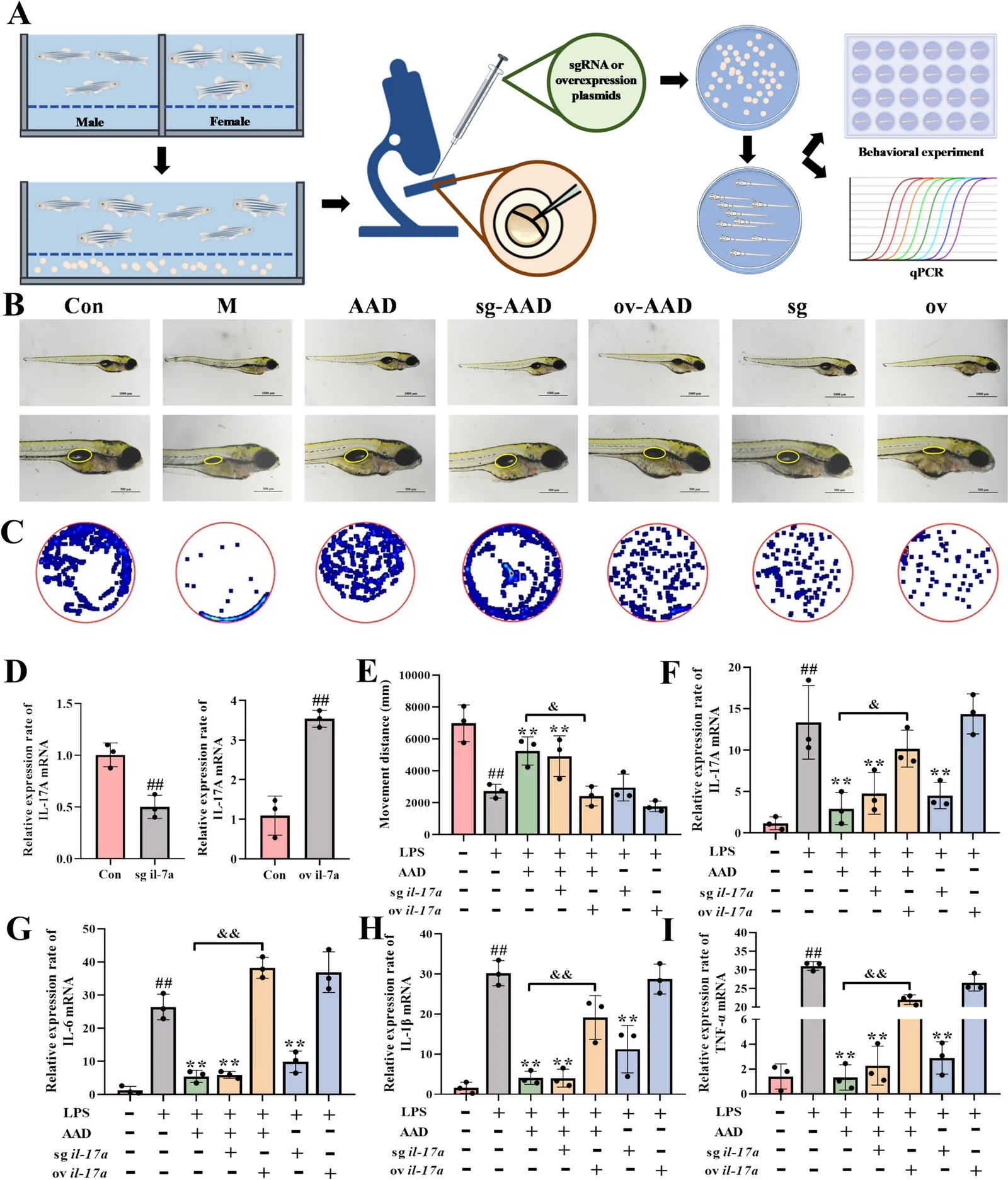
The effect of AAD on LPS-induced injury in Zebrafish. **A** Zebrafish experimental flowchart. **B** Images of zebrafish in bright field. **C**, **E** Heat maps of zebrafish movement and their quantitative results. **D** Silencing and overexpression of IL-17A gene. **F** Expression of IL-17A RNA in each group of zebrafish. **G**–**I** Expression of IL-6, IL-1β and TNF-α RNA in each group of zebrafish. The data were expressed as the mean ± SD, n = 3. ^#^*P* < 0.05, ^##^*P* < 0.01 compared with the Con group. ^*^P < 0.05, ^**^P < 0.01 compared with the M group. ^$^*P* < 0.05, ^$$^*P* < 0.01 compared with the AAD group

To further verify whether AAD exerts its effect through IL-17A, we used a zebrafish model and silenced and overexpressed the IL-17A gene (Fig. 10A). The results showed that the swim bladders of the model group zebrafish significantly decreased, accompanied by pericardial edema and a significant reduction in the swimming distance (Fig. 10B, C, E, *P* < 0.01). After AAD intervention, the swim bladders of the zebrafish recovered, and the edema was significantly alleviated, restoring their swimming ability (*P* < 0.01). PCR results showed that the levels of inflammatory factors in the model group zebrafish significantly increased, and the RNA level of IL-17A significantly increased (Fig. 10F–I, *P* < 0.01). AAD significantly inhibited the release of inflammatory factors and the expression of IL-17A (*P* < 0.01). However, these phenomena were reversed after overexpression of the IL-17A gene, and there was a further improvement trend after silencing the IL-17A gene (*P* < 0.05, *P* < 0.01). The above results indicate that AAD improves LPS-induced zebrafish damage through IL-17A target.

## Discussion

Acute lung injury (ALI) is a pulmonary inflammatory disease triggered by various factors such as infection and trauma [27]. Its core pathological features include increased permeability of pulmonary microvessels, damage to the alveolar epithelial barrier, and uncontrolled inflammatory response [28]. Lipopolysaccharide (LPS), as the main pathogenic component of Gram-negative bacteria, is a classic trigger for ALI [29]. At present, there are no effective therapeutic methods for ALI in clinical practice [30]. The main approach is to provide symptomatic and supportive treatment. Therefore, developing new therapeutic drugs with anti-inflammatory activities holds significant clinical value. Traditional Chinese medicine, due to its unique advantages of multiple components, multiple targets and low side effects, has shown great potential in the prevention and treatment of ALI [31]. In the early stage of the laboratory research, a large number of compounds were extracted and isolated from *Alisma orientale*. Moreover, in vitro experiments demonstrated that these compounds could significantly improve the damage of Beas-2B cells [13, 14]. As AAD is a compound isolated from Alisma orientale, its biological activity has not yet been systematically elucidated. Therefore, this study explored the therapeutic effect of AAD on ALI for the first time by establishing an ALI model induced by LPS, which provided experimental basis for the development of active components of traditional Chinese medicine for clinical treatment of ALI.

LPS directly damages the integrity of the pulmonary vascular endothelium and alveolar epithelial barrier, resulting in a significant increase in vascular permeability and causing pulmonary edema [32]. At the same time, it is accompanied by typical pathological damages such as inflammatory cell infiltration and destruction of alveolar structure [33]. AAD significantly reduced the wet-to-dry ratio and water content of the model group mice. Moreover, the pathological staining results showed that AAD could significantly alleviate the inflammatory infiltration caused by LPS and restore the alveolar structure. In addition, LPS can trigger the ROS burst [34]. Excessive ROS will inhibit the function of the antioxidant defense system, forming a vicious cycle of "oxidative damage—decreased antioxidant capacity" [35]. More importantly, the imbalance of oxidative stress can also activate the apoptotic pathway, promoting cell apoptosis and further damaging the structural integrity of lung tissue, ultimately leading to abnormal lung function [36]. The experimental results showed that after LPS-induced modeling, the mice in the model group exhibited abnormal oxidative stress levels, significantly increased apoptosis levels in lung tissue, and significant decreases in MV, PEF, PIF, and f. AAD significantly improved the imbalance of oxidative stress, inhibited the level of apoptosis level in lung tissue, and restored lung function. The above results preliminarily indicate that AAD can alleviate lung tissue damage and improve lung function in LPS-induced ALI mice.

Excessive ROS directly disrupts the metabolic regulatory network of lung tissue cells, and simultaneously causes abnormal serum metabolic profiles through systemic inflammation [37]. The results of lung tissue and serum metabolomics showed that the metabolic profile of the model group mice was abnormal. After AAD intervention, the metabolic profile tended to be similar to that of the control group. It mainly involved the arachidonic acid metabolic pathway. The arachidonic acid metabolism was closely related to the level of inflammation. Leukotrienes can activate RORγt and thereby increase the transcriptional level of IL-17. In addition, arachidonic acid can directly activate NF-κb and thereby promote the release of inflammatory factors [38]. Further analysis of the GEO database revealed that the IL-17 and NF-κB signaling pathways play a significant role in the development of ALI. And IL-17 can directly activate the NF-κB signaling pathway [39]. After the NF-κB pathway is activated, it will reverse promote the activation of the IL-17 pathway through transcriptional regulation, forming a positive feedback loop of "IL-17–NF-κB–IL-17" for inflammation. Among the IL-17 family, IL-17A, IL-17C, IL-17E and IL-17F are the most common [40]. Further molecular docking and PCR results indicate that AAD may regulate the NF-κB signaling pathway through IL-17A. The WB and immunofluorescence results showed that AAD significantly inhibited the protein expression of IL-17A and p-NF-κB-P65. The activated NF-κB can recruit and activate macrophages and neutrophils [41]. The activated macrophages will release pro-inflammatory factors such as IL-6, IL-1β and TNF-α [42]. And these inflammatory factors will in turn activate the NF-κB pathway, further intensifying the inflammatory level [43]. The results from the flow cytometry and the kits showed that the levels of macrophages, neutrophils and inflammatory factors in the ALI mice were significantly increased. AAD was able to inhibit the activation of macrophages and neutrophils, as well as the release of inflammatory factors. Similarly, in the lung organoid model, AAD significantly improved the LPS-induced damage to lung organoids and inhibited the levels of inflammatory factors. The above results indicate that the protective effect mediated by AAD may be achieved by inhibiting the IL-17A/NF-κB signaling pathway.

To verify whether AAD exerts its efficacy through IL-17A/NF-κB, we established a LPS-induced cell injury model in Beas-2B cells, and conducted the verification by combining the antagonist (Brodalumab) and the agonist (SR0987) of IL-17. The results showed that AAD significantly improved the levels of apoptosis and oxidative stress induced by LPS, inhibited the expression of proteins related to the IL-17A/NF-κB pathway and the release of inflammatory factors, and exhibited the same effect as the IL-17 antagonist. Moreover, after the addition of the IL-17 antagonist, the effect was further enhanced in some indicators. However, after the addition of the IL-17 agonist, the effect of AAD was reversed. In addition, we also used the zebrafish model to verify the results by silencing and overexpressing the IL-17A gene respectively. The results showed that after LPS-induced modeling, zebrafish exhibited a reduction in swim bladder size and pericardial edema, and the levels of inflammatory factors and IL-17A RNA increased significantly. After the AAD intervention, the LPS-induced damage was significantly restored and the release of inflammatory factors was inhibited. However, the efficacy of AAD was reversed after overexpression of the IL-17A gene. These results further indicate that AAD may improve LPS-induced damage through the IL-17A/NF-κB signaling pathway.

## Conclusion

This study established in vivo models in mice and in vitro models of lung organoids to demonstrate that AAD can effectively alleviate ALI induced by LPS. By creating cell models and utilizing IL-17 pathway agonists and antagonists, as well as establishing zebrafish models for IL-17A gene silencing and overexpression experiments, we further validated that AAD exerts anti-inflammatory effects through the IL-17A/NF-κB signaling pathway. This research reveals a novel mechanism by which AAD combats ALI, providing substantial experimental evidence for its potential development as a candidate drug for the treatment of ALI.

## Supplementary Information

Supplementary material 1: Figure S1 The structural identification of compound AAD. A. The ^1^H-NMR spectrum of compound AAD. B. The ^13^C-NMR spectrum of compound AAD. C. The DEPT 135 spectrum of compound AAD. D. The ^1^H-^1^H COSY spectrum of compound AAD. E. The HSQC spectrum of compound AAD. F. The HMBC spectrum of compound AAD. G. The NOESY spectrum of compound AAD. Figure S2 The effect of AAD on metabolic levels in the serum of ALI mice. A. PCA score plots, OPLS-DA scatter plots, S-plot scatter plots, and 200 permutation test plots for each group of serum samples in the positive and negative ion modes. B. Heatmap of cluster analysis for potential biomarkers in serum. C-D. Quantitative expression of metabolites related to the arachidonic acid metabolic pathway. E. Enrichment analysis of biomarker metabolic pathways. F. Correlation analysis between metabolites associated with arachidonic acid metabolism and inflammatory factors. The data were expressed as the mean ± SD. ^#^*P*<0.05, ^##^*P*<0.01 compared with the Con group. ^*^P<0.05, ^**^P<0.01 compared with the M group. Figure S3 IL-17C protein prediction structure. Figure S4 The sequence of IL-17A gene silencing and overexpression. A. IL-17A gene map. B. Vector V013 pcDNA3.1map. C. IL17Ain pcDNA3.1.

## Data Availability

No datasets were generated or analysed during the current study.

## Abbreviations

AAD: Alisol A 23,24-diacetate
ALI: Acute lung injury
ARDS: Acute respiratory distress syndrome
Bro: Brodalumab
BALF: Bronchoalveolar lavage fluid
ELISA: Enzyme-linked immunosorbent assay
GSH-Px: Glutathione peroxidase
H&E: Hematoxylin and eosin
LPS: Lipopolysaccharide
MDA: Malondialdehyde
MV: Minute volume
PEF: Peak expiratory flow
PIF: Peak inspiratory flow
f: Respiratory rate
Res: Resveratrol
ROS: Reactive oxygen species
SR: SR0987
T-SOD: Total superoxide dismutase
