# Supplementary material for: Alisol A 23,24-diacetate improves LPS-induced acute lung injury through the IL-17A/NF-κB signaling pathway
Source: Chin Med. 2026 Aug 24;21:213. doi: 10.1186/s13020-026-01487-w (PMC13501606; doi:10.1186/s13020-026-01487-w)
Supplement: Supplementary file 1 [file 13020_2026_1487_MOESM1_ESM.docx]

**Supplementary material**

**S1**

The structural identification of compound AAD. We recognize the current limitations in the quality control of AAD. In subsequent research, we plan to take all these factors into full consideration and will conduct comprehensive quality evaluation, endotoxin detection, and residual solvent analysis to further refine and standardize the experimental materials.

Figure S1 The structural identification of compound AAD

A. The ^1^H-NMR spectrum of compound AAD (500 MHz, CD_3_OD). B. The ^13^C-NMR spectrum of compound AAD (125 MHz, CD_3_OD). C. The DEPT 135 spectrum of compound AAD. D. The ^1^H-^1^H COSY spectrum of compound AAD. E. The HSQC spectrum of compound AAD. F. The HMBC spectrum of compound AAD. G. The NOESY spectrum of compound AAD.

**S2**

Further serum metabolomic results demonstrated that in both positive and negative ion modes, consistent with the lung tissue metabolomics findings. PCA and OPLS-DA indicated that AAD treatment significantly reversed the serum metabolic profile of ALI mice (Fig. S2A). Similarly, the quantitative analysis of the differential metabolites and the cluster analysis results indicated that AAD improved the metabolic disorders in mice induced by LPS (Fig. S2B-D, *P*<0.05 or *P*<0.01). Metabolic pathway enrichment analysis showed that AAD primarily participated in arachidonic acid metabolism (Fig. S2E). Correlation analysis indicated that arachidonic acid-related metabolites were associated with inflammatory factor indicators (Fig. S2F). These results suggest that AAD may alleviate LPS-induced ALI by modulating arachidonic acid metabolism, which serves as a key pathway regulating inflammatory levels.

Figure S2 The effect of AAD on metabolic levels in the serum of ALI mice

A. PCA score plots, OPLS-DA scatter plots, S-plot scatter plots, and 200 permutation test plots for each group of serum samples in the positive and negative ion modes. B. Heatmap of cluster analysis for potential biomarkers in serum. C-D. Quantitative expression of metabolites related to the arachidonic acid metabolic pathway (n=4). E. Enrichment analysis of biomarker metabolic pathways. F. Correlation analysis between metabolites associated with arachidonic acid metabolism and inflammatory factors. The data were expressed as the mean ± SD. ^#^*P*<0.05, ^##^*P*<0.01 compared with the Con group. ^*^*P*<0.05, ^**^*P*<0.01 compared with the M group.

**S3**

Download the IL-17C protein sequence based on the Uniport number (Q9P0M4). And then use AlphaFold3 to predict its dimer structure. If the pLDDT>70, pTM>0.5, and ipTM>0.6, it indicates that the predicted structure is reliable. The protein structure of IL-17C is shown in Figure S3.

Figure S3 IL-17C protein prediction structure

**S4**

The synthesized silent RNA sequences: sgRNA 1: GAGACTTGCGCATTTTTTCA, sgRNA 2: GATGAGGGCGCTCGCAAACT.

The overexpressed sequences are shown in Fig. S4.

Figure S4 The sequence of IL-17A gene silencing and overexpression

A. IL-17A gene map. B. Vector V013 pcDNA3.1(+) map. C. IL17A(NM_001328553.2) in pcDNA3.1(+)
